# Preparation and characterization of morphine gelatine microspheres

**DOI:** 10.1080/15685551.2022.2158571

**Published:** 2022-12-20

**Authors:** Xin Jin, Jun Ji, Yonghai Sun

**Affiliations:** a Anesthesiology Department, the Chinese PLA Air Force Medical Center, Beijing, Hebei, China; b Department of Comprehensive Treatment, the Second Medical Center of the Chinese PLA General Hospital, Beijing, Hebei, China

**Keywords:** Morphine, microspheres, biological availability, analgesia, pharmacokinetics

## Abstract

Morphine is a widely used opioid analgesic. However, standard morphine dosages and administration methods exhibit a short half-life and pose a risk of respiratory depression. Sustained-release microspheres can deliver prolonged efficacy and reduce side effects. We present a new controlled-release morphine gelatine microsphere (MGM) prepared using an emulsification-crosslinking strategy. The gelatine microsphere design improves the bioavailability of morphine. And it not only increases the clinical analgesic efficacy but also the safety of clinical medication through a gradual, sustained release. Besides, we describe MGMs’ preparation, release, pharmacodynamics, and pharmacokinetics. And the drug metabolism pathway. We calculate the release rate of morphine by measuring plasma morphine concentration over time and pharmacokinetic parameters. It optimized the manufacturing process of MGMs, which makes the analgesic effect have a longer duration. MGMs analgesic effect shows dose dependence. After they were administrated, MGMs were released more slowly. Peak concentration was reduced, and the relative bioavailability improved. It even reached 88.84%. Its pharmacokinetic process was consistent with the two-component first-order absorption model. MGMs deliver sustained-release and long-action pharmacokinetics. It shows design goals of improving drug bioavailability, prolonging drug residence time in vivo, and maintaining stable blood drug concentration.

## 1. Introduction

With the gradual maturity of the controlled drug release system, the system has become the first choice for improved drug deliveries [1]. Because it overcomes the shortcomings of conventional drugs, such as the short half-life of sustained release, high systemic toxicity, and frequent administration [2], and its flexible delivery methods, such as intramuscular, intravenous, and pulmonary inhalation [3].

As a classical opioid analgesic, morphine has been widely used in postoperative analgesia and chronic cancer pain treatment [3]. The most commonly used clinical administration method is a single dose or continuous infusion of an analgesic pump, both of which have a good analgesic effect. Some disadvantages were found with the above administration, such as a short half-life (1.7–3 h) with a single dose [4]. Repeated administration leads to high plasma concentration and respiratory depression. An Analgesic pump administration results in more stable blood levels, less likely to cause respiratory depression [5]. However, due to its external equipment, it is not convenient to move. So morphine sustained-release formulations have been designed [6–8] with both fast-release and sustained-release components. There are few reports on locally administered controlled-release morphine [6,8,9].

Currently, there are mainly two kinds of morphine sustained-release preparations widely used in the clinic. At first, oral sustained-release preparations, such as MethcontinR (Mengdi Pharmaceutical), were invented and applied to clinical practice, benefiting many cancer pain patients [9]. However, clinical experience and literature reports [10,11] have shown that long-term oral administration of these drugs can cause severe gastrointestinal irritation reactions in patients. For example, constipation, nausea, vomiting, and other gastrointestinal symptoms are common after prolonged use [11]. The symptoms mentioned above can be relieved by administering a controlled-release suppository via the rectal route. As an alternative method of treating cancer pain, the morphine sulfate suppository MoraxenR has become popular [12]. These complications are excruciating for doctors and patients. Subsequently, as an alternative method of treating cancer pain, the morphine sulfate suppository Moraxen ^R^ has been used. It can alleviate not only cancer pain but also the above gastrointestinal symptoms. It acts as a solid embolism into the rectum that melts rapidly due to the temperature of the cavity, and the analgesic efficiency is acceptable. But the drug-containing matrix lacks adhesion, leading to the partial outflow or transfer to the formation depth and absorption through the colon endothelium to produce the first-pass effect [13]. There is no doubt that this process reduces the bioavailability of the drug.

It is well known that the way to improve drug bioavailability is to apply drugs locally or directly to the affected area, which can enhance the local therapeutic effect and reduce the number of drugs entering the systemic circulation and the loss of first-pass impact [14–16]. The morphine microspheres we designed are a localized slow-release system administered by local injection.

In recent years, more and more studies have been conducted on the drug slow-release system with polymers such as PLA/PLGA as the carrier, which has a longer degradation time and more foreign body reactions in the injection site pathology [17–20]. Gelatin is a natural material readily degraded in vivo and is better biocompatible and more stable in release than gels and polymers [21–23]. Therefore, the gelatin morphine microspheres were designed and tested.

## 2. Materials and methods

### 2.1. Reagents and instruments

Morphine hydrochloride injection (Shenyang No. 1 Pharmaceutical Factory of Northeast Pharmaceutical Group, batch number: 070606), medical gelatine (Chemical Reagent Company of Beijing), span 85, isopropanol (IPA); precision balance (Sartorius Bp211D, Germany), LC-10Avp high-performance liquid chromatograph (Shimadzu, Japan), KQ-50 DB ultrasonic cleaner (Kunshan Ultrasonic Instruments Co. Ltd), RW20 electric mixer (IKA Company), and electric heating constant temperature water-bath (Shazhou Medical Apparatus Factory in Jiangsu Province).

### 2.2. Experimental animals

Sixteen New Zealand rabbits (eight males and eight females) with an average weight of 2.71 ± 0.28 kg were used in this study. The experimental rabbits were graded as the ‘cleanest’ and were provided by the Experimental Animal Centre of Chinese PLA Postgraduate Medical School. They lived in a soundproof animal laboratory at a room temperature of 24–25°C. The daily cycle alternated over 12 h. They could eat and drink freely and were acclimatized to the environment for one week. The experimental animals fasted for 12 h before being drugged and caged. Institutional Animal Care and Use Committee and the PLA Ethics Committee approved the animal study. The approval document is batch Number 2018-X14-10.

### 2.3. Microspheres preparation

According to the orthogonal experimental design, an appropriate amount of gelatine (20%, 25%, 30%) was weighed and dissolved in distilled water. Simultaneously, a proper amount of morphine was considered and dried for three hours at a constant temperature (105°C) (Table 1). Next, morphine and gelatine were combined in a ratio of 1:2,1:2.5, and 1:3 in a water bath at 55°C(sample A). Liquid paraffin and sorbitan trioleate (Sigma)mixed well (Sample B). Sample A was slowly dropped into sample B with agitation to even mixing. The sample was emulsified and cooled on ice. And then, the filtrate was cleaned four times with acetone and diethyl ether, dried under a vacuum, classified, weighed, and packaged separately until stored. Thermal sterilization was performed at 60°C. The method used to prepare blank microspheres was the same as the way, except morphine was not included. There are specific steps in the supplement material.

**Table 1.** Orthogonal experiment factors and levels.

Factor	A Gelatine (%)	B (Feed ratio = morphine:gelatine)	C (Emulsion: Span 85 %)	D Stirring speed (rpm)
Level 1	20	1:2	1	1000
Level 2	25	1:2.5	2	800
Level 3	30	1:3	3	700

Orthogonal experiment: Based on a preliminary test and single-factor inspection, we chose four target variables: gelatine concentration, feed ratio, Span 85 concentration, and stirring speed. Three levels were set for each factor (Table 1).

## 3. Microsphere characterization

### 3.1 Morphology

Microspheres were coated on a slide, dispersed with an appropriate amount of double distilled water, observed, and imaged at a magnification of 400× using a scanning electron microscope.

(1) $$Span\left(S1\right)=\frac{D90-D10}{D50}$$

PS: D90, D50, and D10 indicate the numbers of microspheres with diameters of less than 90%, 50%, and 10% of all the microspheres, respectively.

### 3.2 Stability

We investigate the sample stability by placing samples in stable conditions, such as intense light and high temperature and humidity. Then, we need to recheck the pieces to find their changes in content and morphology.

*High-temperature experiments* Morphine gelatin microspheres (MGMs) were spread as a 5 mm thin layer in a petri dish and placed in an electric thermostatic incubator at 20°C,30°C,40°C,50°C and 60°C for ten days.

*High humidity tests* The test products were placed in a constant humidity closed container at 25°C, relative humidity (RH) 90 ± 5% for ten days. Samples were taken on the 5th and 10th days for detection. The tested parameters should include hygroscopic weight gain. A constant humidity condition can be achieved by using a constant temperature and humidity box or placing a saturated salt solution under a closed container. According to different humidity requirements, a saturated solution of NaCl (15.5–60°C, RH 75 ± 5%) or KNO3 (25°C, RH 92.5 ± 5%) was selected.

*Illumination experiments* The samples were placed in a lightbox or another suitable light container for ten days under illuminance (4500 ± 500) lx. Samples were taken on the 1,3,5,7 and 10 days for testing.

### 3.3 Assessment of microspheres

Assessment of microspheres was based on sum value (S) of span (S1), yield (S2), encapsulation rate (S3), and drug loading (S4) of microspheres, as follows [2]:

(2) S=−S1+S2+S3+S4

The calculation method for span (S1) is mentioned above under the morphology section in equation (1).

(3) $$Yield\;\left(S2\right)=\frac{wt\;of\;dry\;ingredients\;\left(mg\right)}{wt\;of\;all\;ingredients\;used\;\left(mg\right)}\times 100\%$$

(4) $$Encapsulation\;rate\left(S3\right)=\frac{wt\;of\;entrapped\;morphine}{wt\;of\;morphine\;used}\times 100\%$$

(5) $$Drug\;Loading\left(S4\right)=\frac{wt\;of\;entrapped\;morphine}{wt\;of\;microspheres\;used\;for\;dosing}\times 100\%$$

### 3.4. Characteristics of drug release in vitro

MGMs’ in vitro release curves were fitted by zero-order, first-order, Higuchi equation, and Ritger-Peppas index model. The equations for each released model are given below.

(6) $$Zero\;\;Orderkt=\frac{M^{t}}{M^{\infty }}$$

(7) $$First\;order-kt=\ln\left(\frac{1-M^{t}}{M^{\infty }}\right)$$

(8) $$Higuchikt^{1/2}=\frac{M^{t}}{M^{\infty }}$$

(9) $$Ritger-Peppasindexkt^{n}=\frac{M^{t}}{M^{\infty }}\;$$

Here t is the release time, k is a constant, M^t^ is the cumulative release at time t, M ∞ is the cumulative release at time ∞, and M^t^ /M ∞ is the cumulative release percentage.

## 4. Pharmacodynamics – Measurement of cumulative pain scores

### 4.1 Experimental animal grouping

A total of 24 New Zealand rabbits were randomly divided into six groups, with four rabbits in each group. Before the experiment, they fasted for 12 hs and drank water freely. A 4 x 4cm^2^ square was shaved on one side of the midline of their backs. The grouping is shown in Table 2.

**Table 2.** Pharmacodynamics experimental animal grouping.

Group	Effect factor	Drug	Dosing method	Dosage
A	sham (control)	1.5% isoflurane	Inhalation	5 min
B	Blank microspheres (control)	Blank microspheres	Subcutaneous injection	5 mg/kg
C	Morphine hydrochloride	Morphine injection	Subcutaneous injection	1 mg/kg
D	Morphine hydrochloride	Morphine injection	Subcutaneous injection	3 mg/kg
E	Morphine microspheres	MGMs	Subcutaneous injection	5 mg/kg
F	Morphine microspheres	MGMs	Subcutaneous injection	15 mg/kg

The dosage of morphine hydrochloride injection was calculated following its instructions in animal tests. In contrast, the dose of morphine gelatin microspheres was chosen based on their entrapment efficiency. The encapsulation efficiency of produced microspheres was around 20.94%, according to earlier pilot studies. We increased the dose of the morphine gelatin microspheres to equalize the morphine content of the injection of morphine hydrochloride and the microspheres.

### 4.2. Model building

This study adopted a hind plantar incision mode [9,24]. Except for group A, all rabbits were anesthetized by inhaling 1.5% isoflurane for about 2 min. After the left hind paw of the rabbit was disinfected, a 1 cm long incision was made from 0.5 cm of the proximal plantar to the toe under inhalation anesthesia. After the skin was cut, the plantar muscles were lifted and cut longitudinally while keeping the muscle intact and attached. The pressure was applied to stop the bleeding, and the skin was sutured.

### 4.3. Dosing method

After the establishment of the plantar pain model, the rats were subcutaneously injected with morphine as well as the suspension of blank or morphine microspheres in the shaved areas while they were awake.

### 4.4. Pain rating

A rabbit model of incisional pain was given medicine one hour after surgery. The pain behavior was assessed by a cumulative pain score [3,25] which indicated that rabbits were weight-bearing when their hind claws had been pale with pressing. If the rear claws were off the ground, two points were given; if the rear claws were on the ground but not carrying weight, one point was given; if the hind claws were on the ground and carrying weight, zero point was given. The two rear claws were observed on the ground, and the loading condition was observed. Observations were made once every five mins for one min each time; the most common posture in 1 min was taken as the standard, and observation continued for a total of one hour (24 time points). The cumulative pain score was calculated by subtracting the lateral foot score.

Many academics [25–27] use the model of pain caused by chemical and inflammatory stimuli in their research on pain. However, the mechanism and the course of pain differ significantly between the common causes of postoperative pain in clinical patients and the tissue damage and pain brought on by a subcutaneous injection of chemical inflammatory agents.

### 4.5. Pain threshold determination

The rabbits were placed on a shelf in a special cage before preoperative and postoperative administration, and the pain threshold was measured at 5, 10, 20, 30, 40, 50, 60, 90, 120, 180, 240, 300, 360, 420, 480 min after administration. The laser pulse duration was fixed at 25 ms, and the power was increased gradually. The pain threshold was set by placing the pain meter on the soles of the feet and irradiating the soles on both sides to cause the power value of lifting the feet to avoid reflex or neigh. The bilateral mean value was taken as the pain threshold at this point.

## 5. Pharmacokinetics

*The experimental scheme* Sixteen New Zealand rabbits were randomly divided into four groups, with four rabbits in each group. Before the experiment, the rabbits were fasted for twelve hours; they could drink water, and one side of the midline of their back was shaved (4 cm × 4 cm). The following groups were designed: A (morphine hydrochloride group 1 mg/kg), B (morphine hydrochloride group 3 mg/kg), C (morphine microsphere group 5 mg/kg), and D (morphine microsphere group 15 mg/kg). 1 mL blood was taken from each ear marginal vein of the rabbits after the treatment (at intervals of 5,10, 20, 30, 40, 50 min and 1, 1.5, 2, 3, 4, 6, 8, 12, 16 and 24 h), treated with heparin anticoagulant, and centrifuged for 10 min at 2000 rpm. Plasma was collected and stored at −20 ° C.

### 5.1. Statistical methods

The microsphere’s pharmacokinetic data were analyzed by 3P97 software. Other statistical analyses were performed using the SPSS statistical software package for the Social Sciences, version 17.0, SPSS Inc., Chicago, IL, USA). Measurement data were expressed as mean ± standard deviation (SD). One-way analysis of variance (ANOVA) was used to compare the differences between the groups. Two independent-sample t-tests were applied to analyze the difference from the control group. The significance level (α) was set at 0.05.

## 6. Results and discussion

### 6.1. Microspheres production technology

In our study, New Zealand rabbits were selected for the preliminary investigation of the pharmacokinetics of morphine microspheres in vivo. In our pharmacodynamics experiments, a pain model was used that was established by Brennan in 1996 [7]. The pain which is caused by cutting is deep, complex, and persistent. This model is similar to human trauma or postoperative pain and is easy to repeat and reproduce. It is one of the ideal models for studying the treatment of postoperative pain.

The morphine package dose designed for this study was 5–15 mg. The dose design is based on the clinical injection dose of morphine [8]. We chose morphine injections of 1 and 3 mg/kg for observation and comparison in the preliminary experiment. Since the mean drug loading of the morphine microspheres was 20.94%, the equivalent doses of morphine in the microspheres group were about 5 and 15 mg/kg. The preparation method of morphine microspheres in this study is the emulsification cross-linking method. Since many variables are involved in the manufacture of microspheres, the final product quality could alter if some of the significant variables change. We considered the influence of the following factors and calculated the process of microsphere fabrication, which are described as follows.

### 6.2. A single-factor survey of the prescription process

1) Concentration of the emulsifier: With an increase in emulsifier content, the microspheres’ particle size shrank. The synthesis of microspheres with a significant share of particle sizes between 50 um and 80 um was possible when the emulsifier concentration reached 1% to 1.5%. However, the emulsifier concentration is too high to result in microsphere adherence. So an emulsifier at 1% was selected.

2) Concentration: Gelatine content is the critical factor affecting microsphere particle size. When the gelatine concentration was higher, the pellet size was larger; when it was lower, it was the opposite. A significant amount of the microspheres with a particle size of 50 um to 80 um could be generated when the gelatine concentration was 15%–25% (W/V).

1. Feed ratio (morphine: gelatine) Variable feeding ratios resulted in microspheres with different burst release effects, drug loading, and encapsulation efficiency. Each set of data samples’ in vitro dissolving trials served as the basis for the results’ mean values (Table 3).

**Table 3.** Results of different feeding ratio microspheres ($\overset{ˉ}{x}\pm s$).

	1:1.5	1:2	1:2.5	1:3
Loading rate (%)	33.10 ± 2.31	19.36 ± 4.44	21.44 ± 4.11	17.26 ± 5.12
Recovery rate (%)	78.21 ±6.22	79.45 ± 5.32	80.63 ± 6.28	78.34 ± 3.45
Burst effect (%)	56.61 ±5.08	47.31 ± 6.07	44.03 ± 4.10	44.11. ± 5.16

4) Stirring velocity The average microsphere particle size shrank, and the effectiveness of drug encapsulation marginally improved as the stirring speed was increased. A significant share of the microspheres with particle sizes of 50 um to 80 um could be generated when the stirring speed was 700–800 rpm.

The drug loading rate was higher in the 50–80 um microspheres, but there was less ejection of foreign bodies [4].

### 6.3. Orthogonal experiment

The results of drug loading, encapsulation rate, and burst release effect of microspheres with different feeding ratios are given in Table 3.

According to each feeding ratio, we made at least 3–5 batches of microspheres to evaluate each index. Among them, the pellet with a feed ratio of 1:1.5 had the highest burst release, while the pellet with a feed ratio of 1:2.5 had a similar burst release to the pellet with a feed ratio of 1:3, but its drug loading was relatively high. So the above feeding ratio parameters are better for the feed ratio of 1:2.5 microspheres.

(10) $$d_{i}=\frac{Y_{i}-Y_{min}}{Y_{\max}-Y_{min}}$$

(11) $$DF={\left(d1\cdot d2\cdot d3\right)}^{1/3}$$

where Yi is the experimentally measured value of three indexes; Ymax and Ymin are the maximum and minimum acceptable values of Yi, respectively; di is the optimization index of a single index, and DF is the total optimization index of the three indexes (Tables 4–6).

**Table 4.** Acceptable maximum and minimum of the three indicators.

Indicator	Y_min_	Y_max_
50 ~ 80 μm distribution percentage of the microsphere size (%)	40	90
Drug-loading rate (%)	5	25
Recovery rate (%)	95	30

**Table 5.** Orthogonal experiment result.

No.	A	B	C	D	Indicator under study			
					Recovery rate (30%–95%)	Drug-loading rate (5%–25%)	Distribution of size (40%–90%)	DF
1	1	1	1	1	77	17.3	70	0.66
2	1	2	2	2	85	17.1	77	0.74
3	1	3	3	3	81	15.5	83	0.72
4	2	1	2	3	83	15.2	75	0.68
5	2	2	3	1	82	15.4	63	0.59
6	2	3	1	2	79	12.3	71	0.57
7	3	1	3	2	74	10.7	68	0.49
8	3	2	1	3	72	10.1	75	0.50
9	3	3	2	1	69	9.5	60	0.39

**Table 6.** Range analysis for orthogonal experiment.

No.	A	B	C	D
I	58.1	54.5	53.7	51.5
II	55.1	55.2	54.5	54.9
III	49.8	53.4	54.7	56.6
R_j_	8.3	1.8	1.0	5.1

The optimal process conditions for morphine gelatin microspheres were A1B2C1D3, which meant that the gelatin concentration was 20%, the feed ratio was 1:2.5, the emulsifier concentration was 1%, and the stirring speed was 700 rpm. The influencing order of factors affecting the quality of morphine gelatin microspheres was A > D > B > C.

In the preparation of microspheres, each step affects the final properties of the microspheres, and the properties are interdependent. For example, we found that particle size affects the encapsulation efficiency and the release rate during our experiments. Due to the low stirring speed in the process of oil phase volatilization, the particle size of microspheres produced by our previous batch was large, so the release rate and encapsulation rate of microspheres in this batch could have been better. Later, we increased the rate, improving both the encapsulation and release rates. The same findings were found in the literature [9,10].

Different concentrations of gelatin were also used in our previous experiments. It was found that the higher the gelatin concentration, the larger the particle size. The reason is that the increase of gelatin concentration in the organic phase leads to faster deposition at the oil-water interface, which causes rapid solidification of the surface of the microspheres and increases the particle size. In other words, forming a porous structure on its surface affects the drug release rate [10,11]. The same as the volatilizing speed, the gelatin concentration also affects the diffusion rate of the drug. That is to say, all the factors affecting the particle size affect the diffusion rate of the drug [12].

## 7. Microsphere characteristics

### 7.1. Appearance morphology

**Figure 1.**
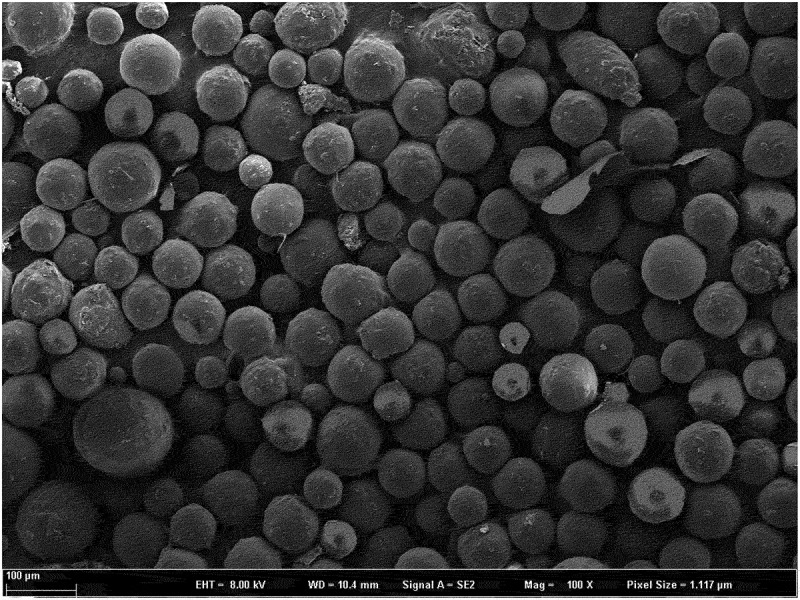
Microscopic photograph of MGMs (×400).

MGMs were seen as light-yellow powders. Using scanning electron microscopy (SEM) (100×), MGMs were round with a smooth surface, no adhesion or little adhesion, good fluidity, and uniform distribution. Their average particle size is 63.11 ± 9.61 µm (Figure 1).

### 7.2. Drug loading and encapsulation rate

HPLC determined the morphine content in the microspheres, and the drug loading and encapsulation rate were calculated according to the formula above. The average drug loading was 20.94%, and the average encapsulation rate was 86.27%. The above results are better than the experimental results under orthogonal conditions, which indicates that the microspheres prepared under this condition are stable and reproducible.

### 7.3. In vitro drug release

**Figure 2.**
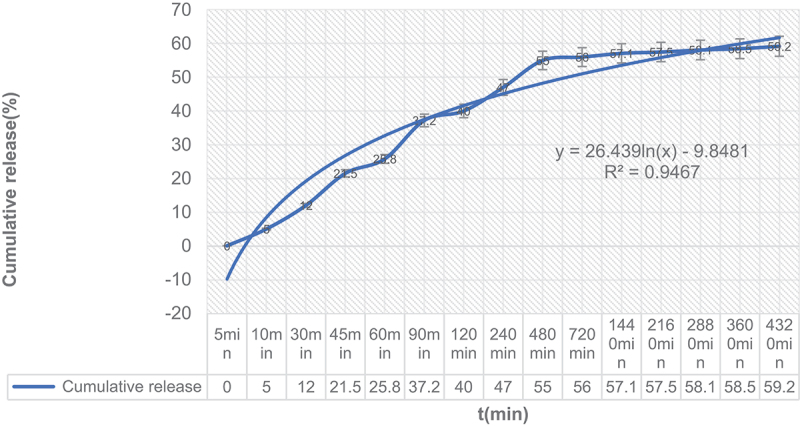
Release profiles of morphine from microspheres *in vitro.*

The *in vitro* release curve of the microspheres prepared by the optimized process is shown in Figure 2. The self-assembled MGMs exhibit a slow-release effect. The microspheres were released rapidly at the initial stage, with a sudden release at four h; the microspheres were released slowly, with more than 68% drug released at 72 h.

The drug release data were fitted by the zero-order kinetic equation, first-order kinetic equation, Higuchi kinetic equation, and the Ritger-Peppas exponential model, respectively (Table 7). The zero-order kinetic equation, first-order kinetic equation, and Ritger-Peppas exponential model could be used to describe microspheres with poor goodness of fit. In contrast, the Higuchi kinetic equation better described the release curve. Therefore, the release of MGMs in vitro conforms to the Higuchi release equation, and the primary mechanism is diffusion.

**Table 7.** *In vitro* release model fit.

model	Equation	R
Zero-order kinetic equation	Q = 0.319 + 0.621 t	0.8568
First-order kinetic equation	ln (1-Q) = −0.522–0.632 t	0.8843
Higuchi kinetic equation	Q = 9.37 t1 /2 + 0. 8659	0.9765
Ritger-Peppas	Q = 7.47 t1.56	0.8311

Drug release from microspheres is a complex process that can be achieved in many ways, such as surface erosion, skeleton diffusion, hydration expansion, dissociation diffusion, disintegration, and desorption [13]. many factors affect drug releases, such as the state of the drug in microspheres, the type and quantity of pellet-forming materials, the size and density of microspheres, the degree of cross-linking, the size and concentration of the drug molecules, the use of additives, the interaction between drug and carrier materials, and the release environment [28,29].

In this study, morphine microspheres released the drug slowly after administration; the peak time was significantly prolonged while the peak concentration was reduced. The plasma concentration-time diagram indicated a burst release process of microspheres in rabbits. The release is then maintained at a relatively low concentration. With the increase in the dose, the sustained release time and concentration of the drug in vivo increased correspondingly. According to the in vitro release characteristics of the microspheres, we speculate that the drug molecules adsorbed to the surface are released rapidly, and the blood drug concentration rises quickly after the microspheres enter the body. The encased drug is then slowly released by diffusion as the microspheres dissolve. The drug release curve was more consistent with the Higuchi drug release equation.

The release of microspheres often has no ideal release environment and release mode. So it takes a lot of work to describe the release process in an equation. It’s a compound way to work. The drug release of most microspheres presents a three-stage release mode: (1) the drug adsorbed on the surface is released rapidly, which is also the leading cause of sudden release; (2) the polymer begins to degrade, the molecular weight decreases continuously, and the drug is released slowly, but the whole system is still insoluble; (3) When the molecular weight of the polymer decreases to a specific value, a large amount of water penetrates the system, leading to the collapse of the skeleton and the release of the drug in large quantities [30,31].

This sudden release effect should be minimized during the fabrication of microspheres. By controlling the particle size of the microspheres and the gelatin concentration of the organic phase, we held the density of holes on the surface of the microspheres. We reduced the amount of sudden release so that the encapsulated drug could be released continuously at a more uniform rate.

Furthermore, the polymer grade and drug loading affected the release rate of microspheres [26,27]. When the degradation rate of the carrier polymer, such as gelatin, is higher than the diffusion rate of the drug, the release rate of the microspheres is mainly related to the degradation rate of the carrier. If the rate of drug diffusion is higher than the rate of carrier polymer degradation, the loaded drug controls its release rate [28].

### 7.4. Stability

We randomly selected three batches of microspheres with a feeding ratio of 1:2.5 to test their stability under different conditions.

*High temperature* Under the light microscope (400x), We found that the burst effect, color, and other characteristics of microspheres at 30°C were not significantly different from those at 20°C. At 40°C, the color of the microspheres was slightly deepened, and the other indexes had no significant change. At 50°C, the color, shape, and burst effect of the microspheres changed, but also some microspheres condensed. The changes in microspheres were more evident at 60°C(Table 8).

**Table 8.** The change of microspheres in high temperature conditions ($\overset{ˉ}{x}\pm s$).

Temperature(°C)	Burst effect (%)	Colour	Deformation	Aggregation
20	44.03 ± 4.10	normal	no	no
30	44.53 ± 3.46	normal	no	no
40	48.71 ±5.73	deepen	no	hardly
50	67.29 ± 8.99	deepen	yes	partial aggregation
60	72.05 ± 11.41	deepen	yes	cluster

*High humidity* Under the RH 90%±5% and RH 75%±5% conditions, the weight of the microspheres increased significantly (Table 9). The hygroscopic color of the particles on the surface of the petri dish deepened, and adhesion occurred.

**Table 9.** Gained weight rate of microspheres under high humidity conditions ($\overset{ˉ}{x}\pm s$).

Gained weight %	5^th^ day	10^th^ day
RH 90%±5%	6.73 ± 3.35	20.47 ± 4.98
RH 75%±5%	5.69 ± 2.10	10.22 ± 3.14

**Figure 3.**
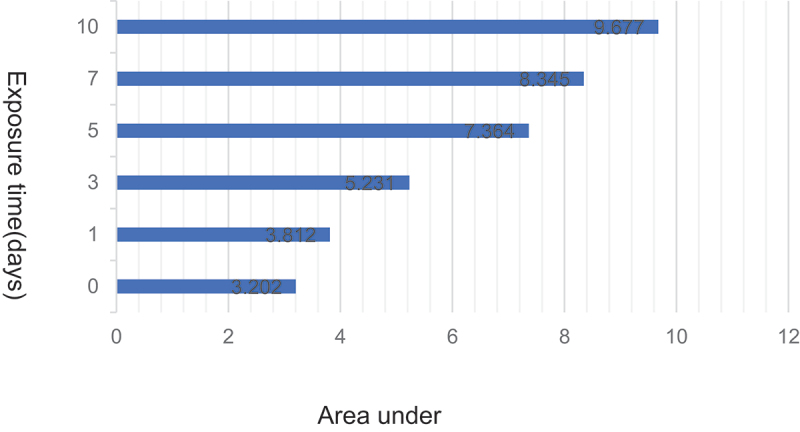
Changes in AUC of the chromatogram of microspheres under high light for 10 days.

*Illumination experiments* morphine is perishable under solid light (4500 lx ± 500 lx). After 1 to 10 days of illumination, the area under the degradation peak curve of the chromatogram of the microspheres increased (Figure 3).

The results of this experiment suggest that morphine gelatin microspheres are suitable for storage below 30 °C in a cool and dry place. The microspheres’ release and degradation rates were mainly affected by temperature and light. And the main effect of humidity on it, we only examined its impact on weight. We thus speculate that poor storage conditions may affect the development of microspheres.

### 7.5. Pharmacokinetics

**Figure 4.**
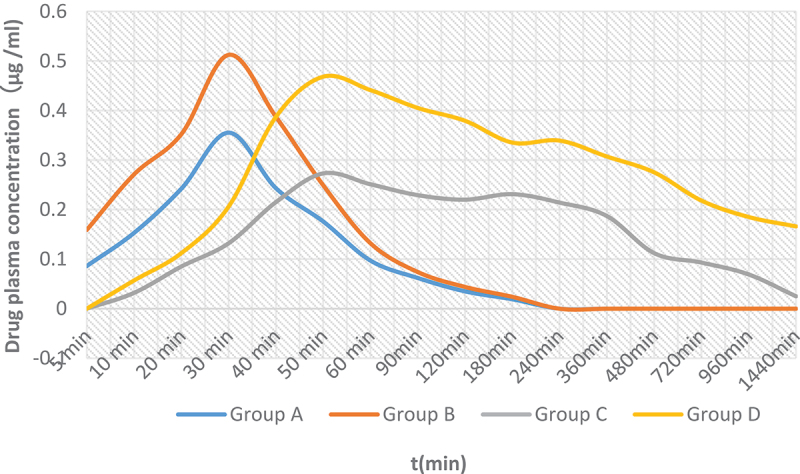
Drug plasma concentration at different times (n = 4).

After subcutaneous injection of morphine, the plasma concentration of rabbits reached the peak at 30 min, then decreased gradually. The average blood drug concentrations of the morphine injection group are shown in Table 10, and data of the morphine microspheres group are shown in Table 11. The variation trends of the drug plasma concentration are shown in Figure 4. The calculation of the pharmacokinetic model was processed by 3P97 pharmacokinetic software. The pharmacokinetic process was consistent with the two-compartment first-order absorption model, and the specific parameters are shown in Table 12. The following groups were designed: A (morphine hydrochloride group 1 mg/kg), B (morphine hydrochloride group 3 mg/kg), C (morphine microsphere group 5 mg/kg), and D (morphine microsphere group 15 mg/kg).

**Table 10.** Drug plasma concentration after subcutaneous morphine injection (n = 4).

Time points	Drug plasma concentration (μg /ml)	
	Group A	Group B
5 min	0.086 ± 0.017 0.153 ± 0.022	0.159 ± 0.070 0.271 ± 0.058
10 min		
20 min	0.242 ± 0.051	0.352 ± 0.095
30 min	0.355 ± 0.102	0.512 ± 0.173
40 min	0.243 ± 0.084	0.387 ± 0.093
50 min	0.176 ± 0.061	0.249 ± 0.071
60 min	0.097 ± 0.025	0.132 ± 0.057
1.5 h	0.062 ± 0.033	0.074 ± 0.033
Two h	0.035 ± 0.011	0.044 ± 0.018
Three h	0.019 ± 0.013	0.024 ± 0.011
Four h	undetected	undetected
Six h	undetected	undetected
Eight h	undetected	undetected
12 h	undetected	undetected
16 h	undetected	undetected
24 h	undetected	undetected

**Table 11.** Drug plasma concentration after subcutaneous injection of MGMs ($\overset{ˉ}{X}\pm S$,n = 4).

Time	Drug plasma concentration (μg/mL ± SD)	
	Group C	Group D
5 min	undetected	undetected
10 min	0.032 ± 0.018	0.057 ± 0.020
20 min	0.085 ± 0.034	0.112 ± 0.026
30 min	0.132 ± 0.067	0.206 ± 0.081
40 min	0.215 ± 0.058	0.387 ± 0.057
50 min	0.273 ± 0.042	0.468 ± 0.063
60 min	0.251 ± 0.033	0.441 ± 0.088
1.5 h	0.229 ± 0.016	0.405 ± 0.071
2 h	0.220 ± 0.028	0.379 ± 0.016
3 h	0.231 ± 0.041	0.335 ± 0.024
4 h	0.214 ± 0.016	0.339 ± 0.051
6 h	0.187 ± 0.036	0.307 ± 0.028
8 h	0.112 ± 0.024	0.275 ± 0.033
12 h	0.093 ± 0.011	0.218 ± 0.043
16 h	0.069 ± 0.025	0.185 ± 0.019
24 h	0.025 ± 0.012	0.166 ± 0.047

**Table 12.** Pharmacokinetic parameters ($\overset{ˉ}{X}\pm S$, n = 4).

Parameters	Unit	Group A	Group B	Group C	Group D
t_l/2α_	min	19.26 ± 4.15	21.44 ± 2.82	40.32 ± 5.33	43.64 ± 3.29
t_l/2β_	min	146.31 ± 7.46	143.56 ± 5.21	337.25 ± 10.36	345.51 ± 8.53
C_max_	μg/mL	0.362 ± 0.045	0.529 ± 0.032	0.275 ± 0.021	0.474 ± 0.035
T_max_	min	31.83 ± 2.30	33.21 ± 3.54	55.22 ± 3.69	59.01 ± 8.24
CL	L /h/ kg	0.412 ± 0.107	0.525 ± 0.085	0.235 ± 0.077	0.229 ± 0.094
AUC	μg /L/ h	287.39 ± 36.87	327.51 ± 65.36	1235.04 ± 42.36	1502.45 ± 25.69

Determination of morphine plasma concentration in rabbits (morphine microspheres group) by HPLC

Ps: A (morphine hydrochloride group 1 mg/kg), B (morphine hydrochloride group 3 mg/kg), C (morphine microsphere group 5 mg/kg), and D (morphine microsphere group 15 mg/kg).

**Figure 5.**
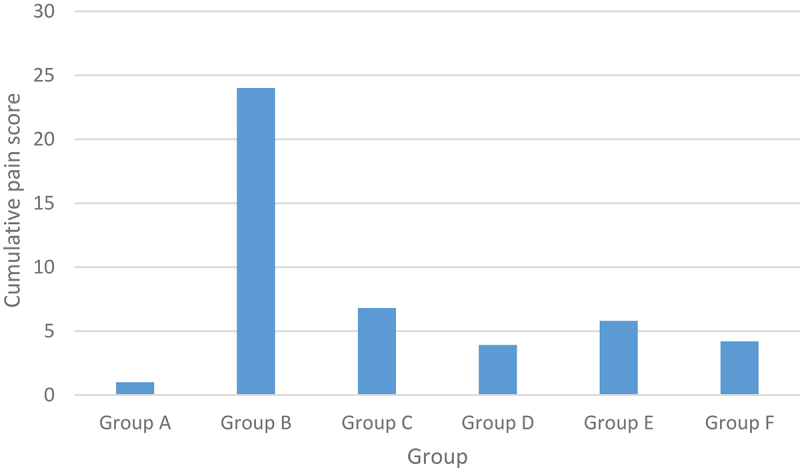
Cumulative pain score.

It can be seen from Tables 11 and 12 that after subcutaneous injection of MGMs in New Zealand rabbits, the microspheres were quickly absorbed into the blood, and the blood concentration could be detected within 10 min. In addition, the greater the dose, the higher the measured ±relationship. Absorption peaks could be observed 50 min after administration (Figure 5). Compared with a morphine injection, the t^1/2^α (distributed half-life) and t^1/2^β (eliminated half-life) of the MGMs were prolonged, AUC (area under the curve of drug duration) increased, T_max_ (peak time) increased, and C_max_ (peak concentration) decreased. The relative bioavailability (F) of MGM capsules versus a morphine injection can be calculated as follows:

(12) $$F=\frac{AUC_{t}\cdot D_{r}}{AUC_{r}\cdot D_{t}}$$

Here D is the administration dose, t is the experimental preparation, and r is the control preparation. F was calculated to be 88.84%.

The data show that the plasma concentration of the morphine injection group reached the peak value 30 min after administration and then decreased rapidly until four h after administration, until the concentration was undetectable. The plasma concentration of the microsphere group produced a peak value at 50 min after administration, which was generated by the burst release of the microspheres. Then the plasma concentration decreased slowly and remained low until 24 h. Statistical analysis showed that the peak concentration of the microsphere group was significantly lower than that of the control group (P < 0.05); t1/2 in the microsphere group was considerably longer than that in the control group (P < 0.01).

Bioavailability (BA) is the degree and rate at which a drug’s active ingredient is released from the product and absorbed into the systemic circulation [32–34]. It is a kinetic property of a drug that needs to be considered when calculating the dose of a non-intravenous medicine (BA is deemed to be 100% by intravenous dosing) [35,36]. When the drug is taken by other means, its bioavailability is reduced due to incomplete absorption and first-pass effects. The area under the drug-hour curve (AUC) represents the total amount of absorption after a single administration, reflecting the degree of absorption of the drug, and can be used to remember the bioavailability of the drug. A large AUC indicates a high bioavailability, and vice versa [34,37]. In this study, compared with the subcutaneous injection of morphine hydrochloride injection, the AUC of New Zealand rabbits increased after a single local subcutaneous injection. The F value reached 88.84%, which was more similar to the effect of intravenous injection of morphine; Tmax time was longer, t1/2α and t1/2β were also relatively prolonged. The above data suggest that MGMs improve the bioavailability of morphine for topical application.

## 8. Pharmacodynamics

### 8.1. Experimental animal grouping

Twenty-four New Zealand rabbits were randomly divided into six groups of four rabbits per group. Shave 4X4cm^2^ on the midline side of the back. The groups were divided as follows: Group A (sham operation group, 1.5% isoflurane inhalation for 5 min without treatment), group B (blank microsphere control group), group C (morphine injection group 1 mg/kg), group D (morphine injection group 3 mg/kg), group E (morphine microsphere group 5 mg/kg), and group F (morphine microsphere group 15 mg/kg).

### 8.2. Cumulative pain score

**Figure 6.**
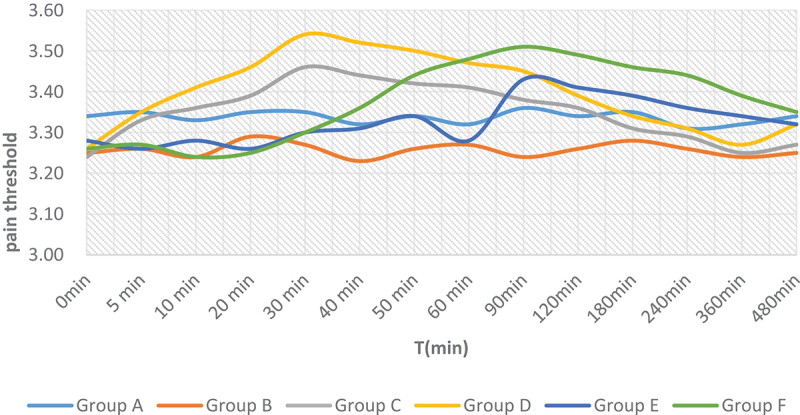
Comparison of pain threshold.

New Zealand rabbits in group A moved as usual, with both hind paws touching the ground and bearing weight. The New Zealand rabbits in group B had upturned feet that could not maintain weight and occasionally landed on the surgically operated side. The cumulative pain scores are shown in Figure 5. The cumulative pain scores of all surgical groups were higher than that of group A (0.312 ± 0.256 P < 0.01); The cumulative pain scores of each administration group were lower than that of group B (21.204 ± 1.315 P < 0.01);

There was no significant difference in cumulative pain scores between groups C and E, D and F; The cumulative pain scores in groups D and F were significantly lower than those in groups C and E (P < 0.01). As can be seen from Figure 6, morphine gelatine microspheres can effectively reduce the cumulative pain score, similar to morphine hydrochloride injection. The cumulative pain score decreased with the increase in dosage, indicating that the analgesic effect of morphine gelatine microspheres was dose-dependent.

Ps: Group A (sham operation group, 1.5% isoflurane inhalation for 5 min without treatment), group B (blank microsphere control group), group C (morphine injection group 1 mg/kg), group D (morphine injection group 3 mg/kg), group E (morphine microsphere group 5 mg/kg), and group F (morphine microsphere group 15 mg/kg).

### 8.3. Pain threshold

The pain threshold of group A was significantly higher than that of group B at all times. The pain threshold of group C and GROUP D began to increase 5 minutes after subcutaneous injection, peaked at 30 minutes, and lasted until 120 minutes after subcutaneous injection. The pain threshold of groups E and F began to increase 30 min after subcutaneous injection, peaked at 90 min, and lasted until 360 min after subcutaneous injection. The pain thresholds of groups D and F were significantly higher than those of groups C and E (figure 7 and Table 13). As can be seen from Figure 6, morphine gelatin microspheres and morphine hydrochloride injection can enhance the pain threshold. And the pain threshold increased gradually with the increase of dose.

**Table 13.** Comparison of pain threshold($\overset{ˉ}{X}\pm S$,n = 4).

Time points	Group A	Group B	Group C	Group D	Group E	Group F
0 min	3.34 ± 0.03	3.25 ± 0.05	3.24 ± 0.04	3.26 ± 0.03	3.28 ± 0.04	3.26 ± 0.04
5 min	3.35 ± 0.04	3.26 ± 0.08	3.33 ± 0.03	3.35 ± 0.05	3.26 ± 0.07	3.27 ± 0.03
10 min	3.33 ± 0.04	3.24 ± 0.03	3.36 ± 0.05	3.41 ± 0.04	3.28 ± 0.05	3.24 ± 0.06
20 min	3.35 ± 0.03	3.29 ± 0.07	3.39 ± 0.08	3.46 ± 0.07	3.26 ± 0.06	3.25 ± 0.09
30 min	3.35 ± 0.06	3.27 ± 0.06	3.46 ± 0.06	3.54 ± 0.05	3.30 ± 0.04	3.30 ± 0.05
40 min	3.32 ± 0.07	3.23 ± 0.05	3.44 ± 0.04	3.52 ± 0.04	3.31 ± 0.06	3.36 ± 0.04
50 min	3.34 ± 0.04	3.26 ± 0.04	3.42 ± 0.06	3.50 ± 0.06	3.34 ± 0.05	3.44 ± 0.02
60 min	3.32 ± 0.06	3.27 ± 0.03	3.41 ± 0.07	3.47 ± 0.03	3.28 ± 0.04	3.48 ± 0.06
90 min	3.36 ± 0.05	3.24 ± 0.05	3.38 ± 0.04	3.45 ± 0.05	3.43 ± 0.05	3.51 ± 0.03
120 min	3.34 ± 0.06	3.26 ± 0.05	3.36 ± 0.05	3.39 ± 0.06	3.41 ± 0.03	3.49 ± 0.05
180 min	3.35 ± 0.04	3.28 ± 0.06	3.31 ± 0.04	3.34 ± 0.07	3.39 ± 0.06	3.46 ± 0.04
240 min	3.31 ± 0.05	3.26 ± 0.03	3.29 ± 0.06	3.31 ± 0.05	3.36 ± 0.04	3.44 ± 0.05
360 min	3.32 ± 0.06	3.24 ± 0.05	3.25 ± 0.03	3.27 ± 0.04	3.34 ± 0.05	3.39 ± 0.07
480 min	3.34 ± 0.05	3.25 ± 0.04	3.27 ± 0.05	3.32 ± 0.08	3.32 ± 0.06	3.35 ± 0.03

The analgesic effect of morphine injection appeared earlier (5–120 min after administration), while the analgesic effect of morphine gelatin microspheres was later and lasted longer (30 min-480 min after administration and lasted longer). The above characteristics determine that morphine injection is more suitable for early immediate analgesia in acute pain, such as intraoperative analgesia under general anesthesia or auxiliary analgesia for wound debridement under local anesthesia. Morphine microspheres are more suitable for postoperative local analgesia.

In general, when the patient has just been extubated and sent to the ward, the patient still has some analgesic effect of opioids in the body. Because the anesthetic in their body is not completely metabolized until 2 hours later. However, after the drug is completely metabolized, the patient will develop hyperalgesia in the surgical area, which is more obvious after 3–12 hours [25,38].

At present, many surgeons are used to administer morphine hydrochloride injection into the joint cavity at the end of arthroscopic surgery. It is well known that its analgesic effect reaches its peak at 30 minutes, and its analgesic effect is greatly weakened after 2 hours. In other words, when the patient returned to the ward, morphine injection analgesia began, and the patient’s anesthetic was completely metabolized within 2 hours, and the analgesic effect of morphine injection basically ended. Although patients feel the double analgesic effect in the early stage, their hyperalgesia will be aggravated after the end of analgesia.

Hypothetically, if the surgeon changed the application of morphine microspheres into the joint cavity, the analgesic effect of the microspheres would take effect before the patient had completely metabolized the anesthetic (50 minutes after the end of surgery), and the analgesic effect of the microspheres would last for 6 hours or even longer. The above characteristics of morphine microspheres can make the blood concentration of morphine more stable, improve the analgesic effect, and reduce addiction. It also makes patients more comfortable after surgery, improves the early postoperative exercise rehabilitation rate, and prevents joint ossification [39–41].

In addition, the analgesic effect of morphine microspheres was enhanced as the dose of morphine increased. The pellet release time and drug concentration increased accordingly. However, it is still necessary to be careful not to overdo it

## 9. Conclusion

Gelatin morphine microspheres prepared in this study have better-sustained release and longer analgesic time than the same dose of morphine hydrochloride injection. After the trial, it was found that the blood concentration of morphine in patients was more stable, the analgesic effect was better, the bioavailability of morphine was improved, the addiction was reduced, and the bioavailability was improved.

## References

[1] Gonvers E, El-Boghdadly K, Grape S, et al. Efficacy and safety of intrathecal morphine for analgesia after lower joint arthroplasty: a systematic review and meta-analysis with meta-regression and trial sequential analysis. Anaesthesia. 2021;76(12):1648–1658. 34448492 10.1111/anae.15569 PMC9292760

[2] Zolnik BS, Burgess DJ. Evaluation of in vivo-in vitro release of dexamethasone from PLGA microspheres. J Control Release. 2008;127(2):137–145. 18282629 10.1016/j.jconrel.2008.01.004

[3] Kim K, Bou-Ghannam S, Thorp H, et al. Human mesenchymal stem cell sheets in xeno-free media for possible allogenic applications. Sci Rep. 2019;9(1):14415. 31595012 10.1038/s41598-019-50430-7 PMC6783458

[4] Liu G, Dong L, Lu K, et al. Preparation and in vivo pharmacokinetics of the tongshu suppository. Biomed Res Int. 2016;2016:1691579. 27610366 10.1155/2016/1691579 PMC5004034

[5] Sampath SS, Garvin K, Robinson DH. Preparation and characterization of biodegradable poly(l-lactic acid) gentamicin delivery systems. Int J Pharm. 1992;78(2–3):165–174.

[6] Tao M, Wang J, Pharmacy DO. Preparation optimization of curcumin bovine serum albumin nanoparticles by central composite design and response surface method. China Pharm. 2019;10;1841–1845 .

[7] Brennan TJ, Vandermeulen EP, Gebhart GF. Characterization of a rat model of incisional pain. PAIN. 1996;64(3):493. 8783314 10.1016/0304-3959(95)01441-1

[8] Koning MV, Teunissen A, van der Harst E, et al. Intrathecal morphine for laparoscopic segmental colonic resection as part of an enhanced recovery protocol: a randomized controlled trial. Reg Anesth Pain Med. 2018;43(2):166–173. 29219935 10.1097/AAP.0000000000000703 PMC5794252

[9] Gu B, Burgess DJ. Prediction of dexamethasone release from PLGA microspheres prepared with polymer blends using a design of experiment approach. Int J Pharm. 2015;495(1):393–403. 26325309 10.1016/j.ijpharm.2015.08.089 PMC4609624

[10] Peng X, Yang L, Heng Z, et al. Surface modification by microencapsule coating and grafting to prepare high hydrophilic polytetrafluoroethylene micropowder. React Funct Polym. 2021;167:105029.

[11] Xiao WA, Zhuang DA, Yz A, et al. Effects of lutein particle size in embedding emulsions on encapsulation efficiency, storage stability, and dissolution rate of microencapsules through spray drying. Lwt. 2021;146(7):111430.

[12] Wang D, Fang J, Liu W, et al. Preparation and characterization of Poly(L-lactide-co-glycolide-co-ε-caprolactone)/1,4-Butanediamine modified maleated poly(D,L-lactide-co-glycolide) blend porous films. J Macromol Sci. 2020;59(6):1–11.

[13] George A, Shah PA, Shrivastav PS. Natural biodegradable polymers based nano-formulations for drug delivery: a review. Int J Pharm. 2019;561:244–264. 30851391 10.1016/j.ijpharm.2019.03.011

[14] Ma Y, Wise AK, Shepherd RK, et al. New molecular therapies for the treatment of hearing loss. Pharmacol Ther. 2019;200:190–209. 31075354 10.1016/j.pharmthera.2019.05.003 PMC6626560

[15] Ma X, Li SJ, Liu Y, et al. Bioengineered nanogels for cancer immunotherapy. Chem Soc Rev. 2022;51(12):5136–5174. 35666131 10.1039/d2cs00247g

[16] Hu T, Yang J, Cui K, et al. Controlled Slow-Release Drug-Eluting stents for the prevention of coronary restenosis: recent progress and future prospects. ACS Appl Mater Interfaces. 2015;7(22):11695–11712. 26011753 10.1021/acsami.5b01993

[17] Su Y, Zhang B, Sun R, et al. PLGA-based biodegradable microspheres in drug delivery: recent advances in research and application. Drug Deliv. 2021;28(1):1397–1418. 34184949 10.1080/10717544.2021.1938756 PMC8248937

[18] Ding D, Zhu Q. Recent advances of PLGA micro/nanoparticles for the delivery of biomacromolecular therapeutics. Mater Sci Eng C Mater Biol Appl. 2018;92:1041–1060. 30184728 10.1016/j.msec.2017.12.036

[19] Lai Y, Li Y, Cao H, et al. Osteogenic magnesium incorporated into PLGA/TCP porous scaffold by 3D printing for repairing challenging bone defect. Biomaterials. 2019;197:207–219. 30660996 10.1016/j.biomaterials.2019.01.013

[20] Rocha CV, Gonçalves V, da Silva MC, et al. PLGA-Based composites for various biomedical applications. Int J Mol Sci. 2022;23(4):2034. 35216149 10.3390/ijms23042034 PMC8876940

[21] Al-Nimry S, Dayah AA, Hasan I, et al. Cosmetic, biomedical and pharmaceutical applications of fish gelatin/hydrolysates. Mar Drugs. 2021;19(3):145. 33800149 10.3390/md19030145 PMC8000627

[22] Samatra MY, Noor N, Razali U, et al. Bovidae-based gelatin: extractions method, physicochemical and functional properties, applications, and future trends. Compr Rev Food Sci Food Saf. 2022;21(4):3153–3176. 35638329 10.1111/1541-4337.12967

[23] Ionescu OM, Mignon A, Minsart M, et al. Gelatin-Based versus alginate-based hydrogels: providing insight in wound healing potential. Macromol Biosci. 2021;21(11):e2100230. 34491617 10.1002/mabi.202100230

[24] Keating D. Regulator of Calcineurin 1 (RCAN1) helps coordinate whole body metabolism and limit energy expenditure. Obes Res Clin Pract. 2019;13(3):262.

[25] Hyo-Jeong L, Jae-Ho, Lee, J. H., Lee, E. O., Lee, H. J., Kim, K. H., Kim, S. H., & Kim, S. H. Retraction: substance P and beta-endorphin mediate electro-acupuncture induced analgesia in mouse cancer pain model. J Exp Clin Cancer Res. 2009;28(1)137. 19818140 10.1186/1756-9966-28-137 PMC2765938

[26] Staahl C, Christrup LL, Andersen SD, et al. A comparative study of oxycodone and morphine in a multi-modal, tissue-differentiated experimental pain model. PAIN. 2006;123(1–2):28–36. 16600508 10.1016/j.pain.2006.02.006

[27] Roberts K, Shenoy R, Anand P. A novel human volunteer pain model using contact heat evoked potentials (CHEP) following topical skin application of transient receptor potential agonists capsaicin, menthol and cinnamaldehyde. J Clin Neurosci. 2011;18(7):926–932. 21550252 10.1016/j.jocn.2010.11.015

[28] Wright JC, Burgess DJ. Long acting injections and implants ||. Advances in Delivery Science and Technology. New York: Springer US; 2012:1–11.

[29] Hare JM, Traverse JH, Henry TD, et al. A randomized, double-blind, placebo-controlled, dose-escalation study of intravenous adult human mesenchymal stem cells (prochymal) after acute myocardial infarction. J Am Coll Cardiol. 2009;54(24):2287–2289. 19958962 10.1016/j.jacc.2009.06.055 PMC3580848

[30] Butreddy A, Gaddam RP, Kommineni N, et al. PLGA/PLA-Based Long-Acting injectable depot microspheres in clinical use: production and characterization overview for protein/peptide delivery. Int J Mol Sci. 2021;22(16):8884. 34445587 10.3390/ijms22168884 PMC8396256

[31] Kardos P, Khaletskaya O, Kropova O. Efficacy and safety of Cineole (Soledum) in the treatment of patients with acute bronchitis: results of an open-label randomized clinical phase III study. Clin Phytosci. 2021;7(1). DOI: 10.1186/s40816-021-00319-8.

[32] Zhou Y, Ma Y, Qi C, et al. Superhydrophobic surface based on nano-engineering for enhancing the durability of anticorrosion. Surf Eng. 2020;37(3):1–11.

[33] Ji EC, Kim JS, Choi MJ, et al. Effects of different physicochemical characteristics and supersaturation principle of solidified SNEDDS and surface-modified microspheres on the bioavailability of carvedilol. Int J Pharm. 2021;597(5):120377. 33581270 10.1016/j.ijpharm.2021.120377

[34] Martinez-Zelaya VR, Zarranz L, Herrera EZ, et al. In vitro and in vivo evaluations of nanocrystalline Zn-doped carbonated hydroxyapatite/alginate microspheres: zinc and calcium bioavailability and bone regeneration. Int J Nanomedicine. 2019;14:3471–3490. 31190805 10.2147/IJN.S197157 PMC6524140

[35] Telange DR, Pandharinath RR, Pethe AM, et al. Calcium Ion-Sodium alginate-piperine-based microspheres: evidence of enhanced encapsulation efficiency, bio-adhesion, controlled delivery, and oral bioavailability of isoniazid. AAPS PHARMSCITECH. 2022;23(4). DOI:10.1208/s12249-022-02236-6 35338414

[36] Shenoy PA. Suitability of stimuli evoked hypersensitivities in hind paws as a clinically relevant pain behavioural measure in rat model of walker 256 breast cancer cellinduced bone pain: an overview. American J Pharmacol Pharmacotherapeutics. 2018;5(1). DOI: 10.21767/2393-8862.100014.

[37] Shenoy P. Establishment, optimization and characterization of a rat model of breast cancer induced bone pain. 2018.

[38] Soppimath KS, Kulkarni AR, Aminabhavi TM. Encapsulation of antihypertensive drugs in cellulose-based matrix microspheres: characterization and release kinetics of microspheres and tableted microspheres. J Microencapsul. 2001;18(3):397–409. 11308229 10.1080/02652040010018083

[39] Wang J, Sun H, Sun WT, et al. Efficacy and safety of intrathecal morphine for pain control after spinal surgery: a systematic review and meta-analysis. 2021;25(6) 2674–2684 . 10.26355/eurrev_202103_25431 33829454

[40] Park SY, Choi GS, Park JS, et al. Efficacy and safety of udenafil for the treatment of erectile dysfunction after total mesorectal excision of rectal cancer: a randomized, double-blind, placebo-controlled trial. Surgery (Oxf). 2015;157(1):64–71. 10.1016/j.surg.2014.07.007 25482466

[41] Kan T, Xu J, Xie J. Condensation heat transfer deterioration on superhydrophobic surface with dense nanostructures. J Phys. 2022;2230(1):012027.

